# Engineering a cell wall-deficient protoplast-incorporated hydrogel for infected wound healing

**DOI:** 10.1093/rb/rbag138

**Published:** 2026-06-18

**Authors:** Yan Zeng, Weichao Ding, Xiaohan Zhou, Qishan Li, Junshu Guo, Yingxian Xiao, Rui Zhang, Jiacong Ai, Junyao Deng, Guanmou Li, Xiaolin Cui, Zhenhua Li

**Affiliations:** School of Biomedical Engineering, Southern Medical University, Guangzhou, Guangdong 510515, China; The Tenth Affiliated Hospital, Southern Medical University (Dongguan People’s Hospital), Dongguan, Guangdong 523059, China; School of Biomedical Engineering, Southern Medical University, Guangzhou, Guangdong 510515, China; The Tenth Affiliated Hospital, Southern Medical University (Dongguan People’s Hospital), Dongguan, Guangdong 523059, China; School of Medicine, The Chinese University of Hong Kong, Shenzhen, Guangdong 518172, China; The Tenth Affiliated Hospital, Southern Medical University (Dongguan People’s Hospital), Dongguan, Guangdong 523059, China; Shenzhen Clinical Medical School, Southern Medical University, Shenzhen, Guangdong 518000, China; The Tenth Affiliated Hospital, Southern Medical University (Dongguan People’s Hospital), Dongguan, Guangdong 523059, China; The Tenth Affiliated Hospital, Southern Medical University (Dongguan People’s Hospital), Dongguan, Guangdong 523059, China; Shenzhen Clinical Medical School, Southern Medical University, Shenzhen, Guangdong 518000, China; The Tenth Affiliated Hospital, Southern Medical University (Dongguan People’s Hospital), Dongguan, Guangdong 523059, China; Shenzhen Clinical Medical School, Southern Medical University, Shenzhen, Guangdong 518000, China; The Tenth Affiliated Hospital, Southern Medical University (Dongguan People’s Hospital), Dongguan, Guangdong 523059, China; Shenzhen Clinical Medical School, Southern Medical University, Shenzhen, Guangdong 518000, China; The Tenth Affiliated Hospital, Southern Medical University (Dongguan People’s Hospital), Dongguan, Guangdong 523059, China; Shenzhen Clinical Medical School, Southern Medical University, Shenzhen, Guangdong 518000, China; The Tenth Affiliated Hospital, Southern Medical University (Dongguan People’s Hospital), Dongguan, Guangdong 523059, China; School of Medicine, The Chinese University of Hong Kong, Shenzhen, Guangdong 518172, China; Joint Laboratory of CUHKSZ-Dalian Practical Biotechnology, The Chinese University of Hong Kong, Shenzhen, Guangdong 518172, China; School of Biomedical Engineering, Southern Medical University, Guangzhou, Guangdong 510515, China; The Tenth Affiliated Hospital, Southern Medical University (Dongguan People’s Hospital), Dongguan, Guangdong 523059, China

**Keywords:** infected wound, bacterial protoplasts, photothermal therapy, alginate

## Abstract

Bacteria-infected wounds remain a major clinical challenge due to persistent infection, impaired tissue regeneration and the increasing prevalence of antibiotic resistance. Conventional antibiotic therapies primarily rely on biochemical bactericidal mechanisms and often fail to effectively modulate the wound-healing microenvironment. Here, we report the engineering of a protoplast-incorporated hydrogel for infected wound treatment, in which cell wall-deficient protoplasts derived from *Rhodopseudomonas palustris* (*R. palustris*) serve as a photothermally active antibacterial component. We demonstrate that these protoplasts retain the near-infrared (NIR) photothermal properties of the parent bacteria, enabling localized, non-antibiotic bacterial eradication upon NIR irradiation. Importantly, removal of the bacterial cell wall substantially reduces endotoxin burden, thereby mitigating inflammation-associated risks compared with intact bacteria. To enable localized delivery and retention, the protoplasts are uniformly encapsulated within a sodium alginate hydrogel, which prevents leakage and maintains a moist wound microenvironment. This protoplast-incorporated hydrogel not only achieves effective antibacterial activity but also exhibits a favorable safety profile. Beyond its photothermal bactericidal function, the system promotes cell migration, collagen deposition and angiogenesis, while reducing pro-inflammatory cytokine levels, collectively accelerating wound closure. Overall, this work presents a hydrogel-based, photothermal, non-antibiotic antibacterial platform that integrates endotoxin-reduced bacterial derivatives with microenvironmental modulation, offering a promising strategy for the treatment of bacterial-infected wounds.

## Graphical Abstract

**Graphical Abstract:**
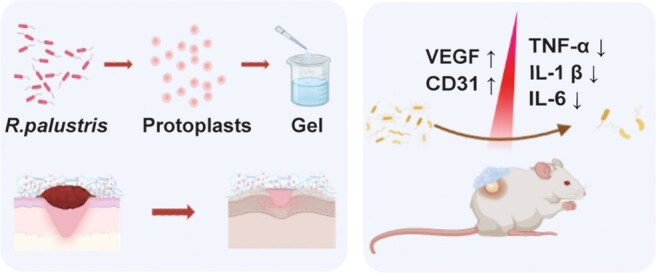

## Introduction

Skin injuries exert a persistent impact on patients’ daily lives, often leading to infections and even mortality [1]. Beyond individual suffering, wounds impose a substantial burden on healthcare systems [2]. Nearly all wounds undergo microbial colonization, which alters the wound microenvironment and may culminate in infection or death [3, 4]. Wound infections are caused by diverse pathogens, and sole reliance on antibiotics fails to fundamentally resolve the issue [5–9]. Thus, developing effective therapies for the recovery of bacterially infected wounds has become an urgent and challenging endeavor. Recently, numerous approaches have been explored for skin wound treatment, including traditional surgical interventions, such as debridement, skin grafting and non-surgical modalities such as dressings and medications [10–12]. Photothermal therapy utilizes the conversion of light into heat to achieve bacterial eradication and promote tissue regeneration and is characterized by high efficiency, precise controllability and minimal risk of drug-resistance development [13, 14]. Current mainstream photothermal antibacterial agents include noble metal materials, metal composites, carbon materials and organic materials [15–17]. However, these agents are limited by poor biocompatibility, high acquisition costs and toxic side effects, hindering their clinical translation [18, 19]. Thus, highly safe and highly stable materials for infected wound healing are urgently needed.

*R. palustris* possesses diverse metabolic pathways that enable flexible utilization of multiple energy and carbon sources [20, 21]. Widely distributed in nature, it represents a readily accessible and renewable biological resource [22, 23]. Notably, we have found photosynthetic bacteria possess Near-infrared (NIR) phototactic ability and photothermal therapeutic effects, rendering them a potential photothermal agent [24–27]. This characteristic further expands *R. palustris*’ utility in wound healing and infection control, offering a promising avenue for biomedical applications [27, 28]. However, endotoxins in the bacterial cell wall are potent stimulants of the inflammatory cascade and a major cause of septic shock, posing substantial risks to human health, which limits their clinical applications [29, 30]. Therefore, the direct therapeutic application of bacteria necessitates careful consideration of their inherent toxicity risks. Current research has primarily adopted two circumvention strategies: the first involves bypassing intact bacteria in favor of bacterially derived functional materials as therapeutic agents [31]; the second employs sophisticated gene knockout techniques to selectively eliminate bacterial virulence factors, thereby fundamentally attenuating their pathogenicity [32].

Bacterial protoplasts, as a class of bacterial derivatives, effectively reduce the toxicity associated with intact bacteria through cell wall removal. Protoplasts are wall-less cells with potential totipotency that can eliminate the adverse effects of endotoxins from the bacterial cell wall, making them more suitable as therapeutic biomaterials [33]. Inspired by this, we designed a protoplast-loaded sodium alginate hydrogel (P@G) dressing to promote the healing of wounds caused by polymicrobial infections. Alginate can form hydrogels in the presence of calcium ions, enabling the stabilization of bioactive compounds [34–36]. It exhibits excellent biocompatibility, high mechanical stability and non-toxicity [37]. These properties make alginate an ideal carrier for wound healing agents. The incorporation of protoplasts endows the dressing with potent photothermal activity and significant antibacterial efficacy. The absence of the cell wall in protoplasts allows photosynthetic pigments to be directly exposed to the NIR light field, resulting in an approximately 25.0% improvement in photothermal conversion efficiency relative to intact bacteria. Concurrently, the release of carotenoids was facilitated, leading to a significant increase in free radical scavenging capacity and a corresponding enhancement of antioxidant performance. A comprehensive evaluation through both *in vitro* and *in vivo* assays demonstrated that the protoplast-incorporated dressing exhibited effective bactericidal activity against gram-negative *Escherichia coli* (*E. coli)* and gram-positive *Staphylococcus epidermidis* (*S. epidermidis*). In a murine model of infected wounds, P@G&N treatment significantly suppressed the expression of pro-inflammatory cytokines Interleukin-1 beta (IL-1β), Tumor necrosis factor-alpha (TNF-α) and Interleukin-6 (IL-6), while promoting cell migration, collagen deposition and angiogenesis, collectively accelerating wound closure. These findings demonstrate that the P@G&N dressing constitutes an innovative wound-healing material with considerable potential for clinical translation.

## Materials and methods

### Extraction of protoplast

Protoplasts were extracted by enzymatic digestion of the bacterial cell wall using lysozyme. To identify the optimal extraction conditions, the lysozyme treatment duration was systematically screened over a range of 0–2.5 h, and the lysozyme concentration was optimized across a range of 0.01–5 mg/mL.

*R. palustris* (BNCC376257) was inoculated into sterile liquid medium and cultured under illumination at 30°C for one week until reaching the logarithmic growth phase. Bacterial pellets were harvested by centrifugation and resuspended in a mixture of 50 mM Tris-HCl buffer (pH 8.0) and 20.0% (w/v) sucrose solution to maintain a hyperosmotic environment. Ethylenediaminetetraacetic acid (EDTA) solution was subsequently added to a final concentration of 50 mM to enhance bacterial susceptibility to enzymatic digestion. Lysozyme was then introduced at a final concentration of 1 mg/mL to hydrolyze the peptidoglycan layer within the bacterial cell wall, thereby inducing cell wall lysis and facilitating protoplast release.

The lipopolysaccharide (LPS) present in the cell wall of *R. palustris* was fluorescently labeled using a Fluorescein isothiocyanate (FITC)-conjugated anti-LPS polyclonal antibody (USCN, Cat. No. ULAB526Ge81). The labeled bacteria were subsequently used as the starting material for protoplast extraction. Both the labeled *R. palustris* and the derived protoplasts were imaged under a fluorescence microscope, and the fluorescence intensity of each group was quantified. The degree of LPS fluorescence signal attenuation was employed as an indicator to confirm the effective removal of the bacterial cell wall during protoplast preparation. Upon confirmation of successful protoplast extraction, residual cell wall debris was removed by centrifugation, and protoplast concentration was determined using a hemocytometer.

The extraction efficiency (Y) of protoplasts was determined by the formula:


$$\text{Y}=({\text{N}}_{1}/{\text{N}}_{0})\times 100\%,$$

with N_1_ representing the total number of viable protoplasts finally obtained and N_0_ the initial total number of bacteria used for extraction.

### Characterization of protoplasts

The surface morphology of *R. palustris* and its derived protoplasts was examined and characterized by scanning electron microscopy (SEM, Apreo 2S, Czech Republic) and transmission electron microscopy (TEM, Talos L120C, Czech Republic). The particle size distribution of protoplasts was subsequently determined by statistical analysis of SEM micrographs.

The endotoxin contents of *R. palustris* and its derived protoplasts were quantified using a Chromogenic Limulus Amebocyte Lysate (LAL) Endotoxin Assay Kit, following the manufacturer’s instructions.

The Ultraviolet-visible absorption spectra of *R. palustris* and its derived protoplasts were recorded over a wavelength range of 300–900 nm using a UV-visible spectrophotometer (PerkinElmer Lambda1050+, UK), with Phosphate-buffered saline (PBS) used as the blank reference for baseline correction.

Equal volumes of *R. palustris* cells and their derived protoplasts were separately resuspended in acetone and extracted for 1 h, after which carotenoid and Bacteriochlorophyll a (BChl a) contents were quantified spectrophotometrically.

The 2,2-Diphenyl-1-picrylhydrazyl (DPPH) and 2,2’-Azino-bis(3-ethylbenzthiazoline-6-sulfonic acid) (ABTS) free radical scavenging capacities of *R. palustris* and its derived protoplasts were evaluated using a DPPH Free Radical Scavenging Capacity Assay Kit (Solarbio, Cat. No. BC4750) and an ABTS Free Radical Scavenging Capacity Assay Kit (Solarbio, Cat. No. BC4775), respectively, in accordance with the manufacturer’s instructions.

### Photothermal properties of protoplasts

Protoplast suspensions at five concentration gradients ranging from 10^5^ to 10^9^ CFU/mL were irradiated with 808 nm NIR light at a fixed power density of 1.5 W/cm^2^ for 8 min to identify the optimal protoplast concentration for photothermal application. Protoplast suspensions at the optimal concentration were subsequently exposed to 808 nm NIR light at varying power densities of 0.5, 1.0, 1.5, 2.0, 2.5 and 3.0 W/cm^2^ for 8 min to determine the optimal irradiation power density. Protoplast suspensions at the optimal concentration and power density were then subjected to multiple cycles of NIR irradiation, with temperature changes recorded after each cycle to evaluate photothermal reusability. Throughout all experiments, temperature variations were monitored in real time using a thermal imaging camera.

Additionally, protoplast suspensions and intact *R. palustris* suspensions at the optimal concentration were irradiated under identical conditions, and their photothermal heating profiles were comparatively analyzed to assess the effect of cell wall removal on photothermal conversion efficiency. The NIR-irradiated protoplast group and the NIR-irradiated *R. palustris* group were designated as P&N and R&N, respectively.

### Analysis of antibacterial activity

Antibacterial activity was evaluated by colony-forming unit (CFU) counting. Gram-positive *S. epidermidis* and gram-negative *E. coli* were selected to assess the broad-spectrum antibacterial efficacy of the test materials. Stationary-phase bacterial suspensions (OD_600_ = 1.0–1.2) were mixed with the test materials at a 1:1 volume ratio and subsequently irradiated with 808 nm NIR light at a distance of 1 cm from the sample surface. The treated bacterial suspensions (50 μL) were uniformly spread onto nutrient agar plates, incubated at 37°C for 18 h and bacterial colonies were then photographed and enumerated.

Bacterial viability was assessed by live/dead fluorescent staining. Following treatment, SYTO 9 and propidium iodide (PI) (1.5 μL each, 1:1 ratio) were added to the bacterial suspensions and incubated in the dark for 15 min. Stained specimens were imaged under an inverted fluorescence microscope (Thermo Scientific EVOS M5000, UK) and quantitatively analyzed.

### Preparation and characterization of P@G

Hydrogels were prepared using sodium alginate (Macklin, Cat. No. C14131552). Calcium chloride solutions at concentrations ranging from 0.2 to 0.7% (w/v) were crosslinked with 2.0% (w/v) sodium alginate solution via ionic crosslinking, and the optimal calcium chloride concentration was identified. Protoplasts or *R. palustris* were incorporated into the sodium alginate solution at the optimal concentration prior to ionic crosslinking to fabricate the protoplast-incorporated hydrogel (P@G) and the *R. palustris*-incorporated hydrogel (R@G), respectively.

A standard curve was first established by correlating the fluorescence intensity of fluorescently labeled protoplasts with their corresponding mass to enable quantitative determination. The encapsulation efficiency (EE) was calculated using the following formula:


EE (%)=(Mi–M)/Mi×100%,

where Mi represents the total mass of protoplasts initially introduced, and M represents the mass of unencapsulated protoplasts determined from the supernatant fluorescence intensity.

To calculate the swelling ratio, the samples were immersed in PBS (pH 7.4) and incubated at 37°C. After reaching the swelling equilibrium point, the samples were carefully taken out. The surface water of hydrogels was wiped off before weighing. The swelling ratio was calculated using the following formula:


$${\text{S}}_{\text{r}}\;(\%)=({\text{W}}_{\text{f}}–{\text{W}}_{\text{i}})/{\text{W}}_{\text{i}}\times 100\%,$$

where W_i_ is the initial weight of the sample prior to water absorption, W_f_ is the final weight after water absorption and Sr is the sample swelling ratio.

To investigate the degradation behavior of P@G, the P@G were placed in PBS solution and incubated on a constant-temperature shaker (100 rpm, 37°C). At pre-set time points, surface moisture was removed with filter paper, followed by precise weighing of the wet hydrogel. The degradation rate was calculated using the following formula:


$$\text{weight remaining}\;(\%)={\text{W}}_{\text{t}}–{\text{W}}_{\text{i}}\times 100\%,$$

where W_i_ denotes the initial weight of the hydrogel sample prior to the degradation assay, and W_t_ means the wet weight measured at each preset time point during incubation.

Blank hydrogel (Gel), R@G and P@G subjected to NIR irradiation were designated as G&N, R@G&N and P@G&N, respectively. The temperature elevation profiles of G&N, R@G&N and P@G&N were recorded using a thermal imaging camera for comparative analysis. P@G&N was subsequently subjected to multiple photothermal irradiation cycles, and SEM characterization was performed before and after irradiation to evaluate the photothermal stability of P@G&N.

### Rheological testing

A rheometer (Anton Paar MCR102e, Austria) was employed to characterize the rheological behaviors (including storage modulus, loss modulus and viscosity) of the prepared hydrogels. Each hydrogel specimen was positioned at the center of the rheometer’s circular measuring plate; after ensuring uniform contact between the sample and the plate, the corresponding experimental parameters were configured to quantify the storage modulus (G′) and loss modulus (G′′), reflecting the viscoelastic properties of the hydrogel.

The specific tests performed are as follows:

Angular frequency test: Conducted at 37°C, with frequency spanning 1–100 rad/s and strain stabilized at 1.0%. Temperature scanning: Performed from 25 to 70°C, under the parameters of fixed 1.0% strain and 10 rad/s angular frequency. Strain test: Performed at 37°C, with the strain varied from 0.1 to 100% and the frequency kept constant at 10 rad/s.

### Biocompatibility evaluation of protoplasts

Red blood cells (RBCs) isolated from the blood of BALB/c mice (5 weeks, body weight: 16 ± 2 g) were diluted with physiological saline to a 5.0% (v/v) suspension. Protoplasts were dissolved in PBS to prepare a set of serial concentrations. Equal volumes of the RBC suspension and protoplast solutions were thoroughly mixed and incubated at 37°C for 1 h. The upper supernatants were harvested after 1000 rpm for 10 min, and to evaluate the extent of hemolysis, its absorbance was quantified at 562 nm (Tecan Infinite E Plex, Switzerland). To determine the hemolysis rate (%), the following formula was used:


$$\text{Hemolysis}\;\text{rate}\;\left(\%\right)=\left[\left({\text{OD}}_{s-}{\text{OD}}_{n}\right)/\left({\text{OD}}_{p-}{\text{OD}}_{n}\right)\right]\times 100\%,$$

where OD_s_ is the test sample OD value, OD_p_ the positive control OD value and OD_n_ is the negative control OD value.

Biocompatibility of protoplasts was assessed using the Live/Dead Cell Staining Kit (Solarbio, China) according to the manufacturer’s instructions. After co-incubation of protoplasts with cells for 4 h, fluorescence images were acquired using confocal microscopy (Zeiss LSM900 with Airyscan 2, Germany).

To assess the proliferation capacity of Human umbilical vein endothelial cell (HUVEC) and Mouse embryonic fibroblast cell line (NIH-3T3) cells, a Cell Counting Kit-8 (CCK-8) assay was employed. The experiment followed the designated experimental treatments. The 96-well plates were seeded with 1 × 10^4^ cells/well (HUVEC or NIH-3T3) and incubated overnight. Protoplast solution (10^8^ CFU/mL) was added and cells were further cultured. At each pre-set time interval, the original culture medium in each well was carefully aspirated and replaced with 100 μL of a 10.0% CCK-8 working solution (diluted in serum-free medium). The cells were incubated with CCK-8 reagent at 37°C in a humidified atmosphere containing 5% CO_2_ for 4 h, after which the absorbance at 450 nm was measured to assess cell proliferation.

To assess the effect of different materials on cell migration, cells were cultured to the logarithmic growth phase and subjected to serum starvation. Cells were then seeded into the upper chambers of Transwell inserts at a density of 1 × 10^4^ cells/mL, while the test materials were placed in the lower compartment. After incubation for 24 h under standard cell culture conditions, the number of migrated cells was counted to evaluate migratory capacity.

To evaluate the effect of materials on angiogenic potential, a 24-well plate was used to load Matrigel, and the Matrigel needed to be solidified for 1 h at 37°C in a 5.0% CO_2_ atmosphere. Subsequently, 200 µL of HUVECs (1 × 10^5^ cells/mL) were added to each well and cultured for 6 h, after which the morphological changes of cells were observed, photographed and recorded.

### *In vivo* antibacterial effects

The experimental Animal Welfare and Ethics Committee of Dongguan People’s Hospital passed the animal experimental protocol (Approval No.: IACUC-AWEC-202410008). Healthy female BALB/c mice (5 weeks, weight: 16 ± 2 g) were randomized into four groups (*n* = 5): PBS, Protoplast, G&N and P@G&N. A 0.8-cm full-thickness circular skin wound was created on the dorsal side of each mouse, and a mixed bacterial suspension (1:1 volume ratio) containing *E. coli* and *S. epidermidis* (100 μL, 10^8^ CFU/mL) was inoculated onto the wound surface. The infection model was considered successfully established when erythema and purulent exudation were observed at 24 h post-infection, and bacterial colonies could be cultured from wound swabs on both *E. coli* and *S. epidermidis* selective plates.

### *In vivo* wound healing

Wounds treated with PBS served as the negative control group. The negative control group and the protoplast group received 0.2 mL of their respective solutions, while hydrogel-treated groups received 0.2 g of hydrogel per wound. Throughout the treatment process, the temperature elevation at the wound site in each group was recorded in real time using a thermal imaging camera. Following treatment, the wound sites were covered and secured with a medical dressing film (Xinhua, 678512630616, China) to prevent displacement of the applied materials. Body weight and wound area of each mouse were measured at predetermined time intervals to monitor therapeutic efficacy and general health status. To calculate the wound closure rate, the following formula was used:


$$\text{Wound closure rate}\;(\%)=[({\text{W}}_{0}–\text{Wn})/{\text{W}}_{0}]\times 100\%,$$

where W_0_ represents the initial wound area on Day 0 and Wn represents the wound area at the sampling time point.

Mouse serum was collected from each group on Day 3 post-treatment, and the concentrations of pro-inflammatory cytokines IL-1β, TNF-α and IL-6, as well as the angiogenic factor vascular endothelial growth factor (VEGF), were quantified by enzyme-linked immunosorbent assay (ELISA) to evaluate the effects of different treatments on the local inflammatory response and angiogenic capacity.

Wound skin specimens were harvested from each group on Days 0, 4, 8 and 12 to assess the dynamic process of epidermal regeneration. The harvested specimens were fixed in 4.0% paraformaldehyde solution at room temperature for 24 h, cleared and embedded in paraffin. Serial sections of 5 μm thickness were prepared and stained with hematoxylin-eosin (H&E) and Masson’s trichrome to evaluate tissue regeneration. In addition, immunofluorescence staining of CD31 and TNF-α was performed to monitor the dynamic changes in angiogenesis and inflammatory levels during wound repair. CD206 immunofluorescence staining was further conducted to evaluate the expression level of CD206 as a marker of M2 macrophage polarization.

### Long-term biosafety evaluation

Protoplasts labeled with a fluorescent marker were incorporated into the hydrogel, which was then applied to murine wound sites. Wound fluorescence intensity was monitored on Days 0, 3, 5 and 7 using *in vivo* imaging. At the same time points, major organs were harvested and fluorescence intensity was measured. The biodistribution and metabolic clearance of the hydrogel *in vivo* were assessed by tracking fluorescence signal attenuation over time.

Following three rounds of treatment, whole blood specimens were collected from all experimental groups on Day 14 of the experiment. Serum concentrations of alanine aminotransferase (ALT), aspartate aminotransferase (AST), blood urea nitrogen (BUN) and creatinine (CREA) were determined to evaluate hepatic and renal function. Serum concentrations of IL-1β, TNF-α and IL-6 were also quantified to assess the systemic inflammatory response. In addition, major organs were harvested on Day 14 of the experiment and processed for H&E staining to assess the long-term systemic biocompatibility of the hydrogel.

### Statistical analysis

Statistical analysis was performed using GraphPad Prism software. All experiments were conducted in five independent replicates, and data are presented as mean ± standard deviation (Mean ± SD). Comparisons between two groups were performed using unpaired Student’s *t*-test, while comparisons among multiple groups were conducted using one-way analysis of variance (one-way ANOVA) or multi-way ANOVA, as appropriate, followed by Dunnett’s multiple comparisons test or Tukey’s multiple comparisons test for *post hoc* analysis. A *P* value of less than 0.05 was considered statistically significant.

## Results

### Preparation and characterization of protoplasts

Protoplasts of *R. palustris* were successfully prepared by enzymatic digestion. Compared with intact bacteria, the derived protoplasts exhibited reduced endotoxin content and enhanced release of bacteriochlorophylls and carotenoids.

**Figure 1:**
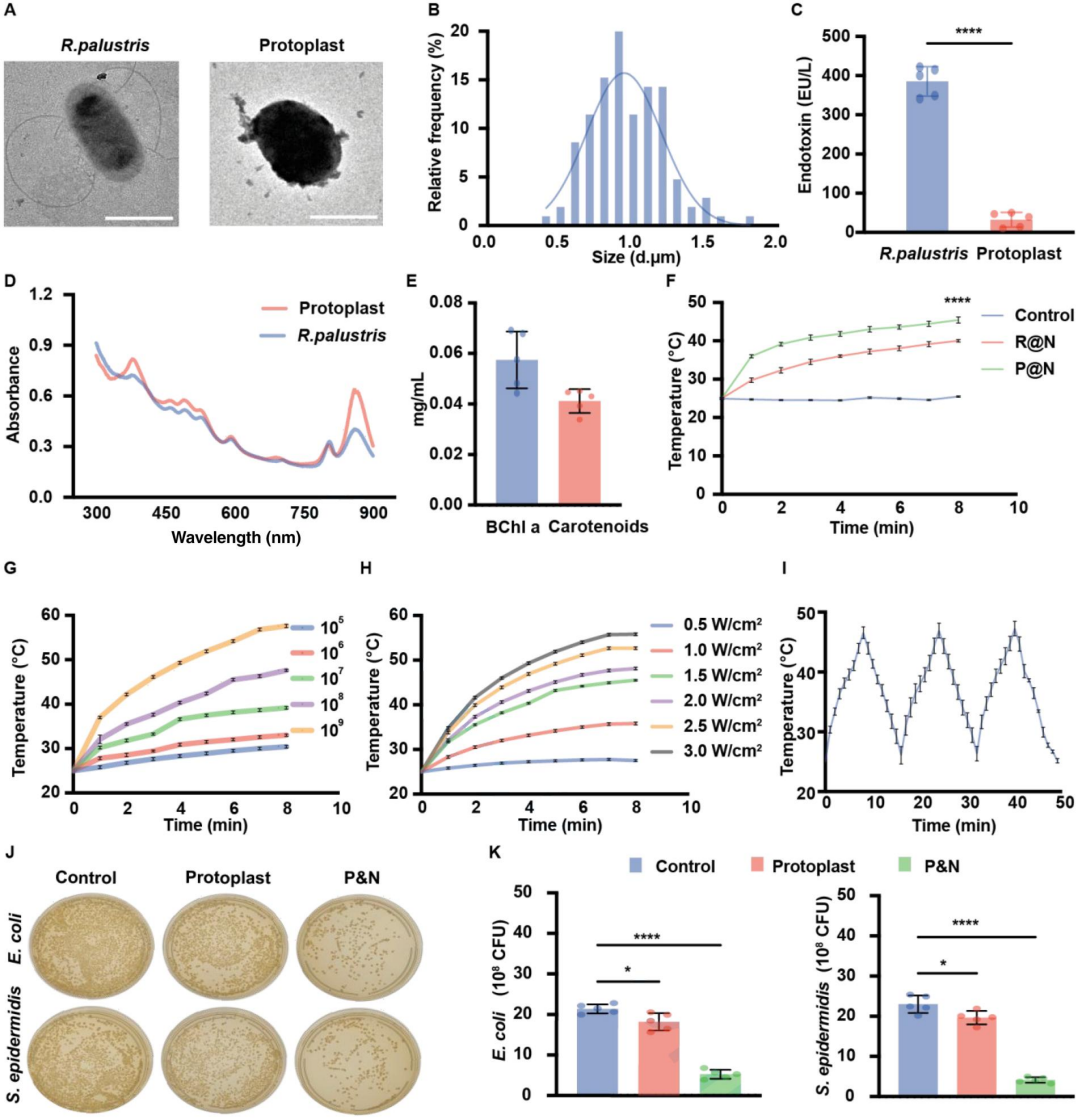
Characterization and photothermal antibacterial performance of *R. palustris* protoplasts. (**A**) SEM micrographs of *R. palustris* and its derived protoplasts. Scale bar: 1 μm. (**B**) Particle size distribution of protoplasts. (**C**) Endotoxin concentrations in protoplast and *R. palustris* suspensions. (**D**) UV-visible absorption spectra of protoplasts and *R. palustris* in the wavelength range of 300–900 nm. (**E**) BChl a and carotenoid contents released into the supernatant from protoplasts suspended in PBS. (**F**) Photothermal heating curves of protoplasts, *R. palustris* and PBS under 808 nm NIR laser irradiation (1.5 W/cm^2^, 8 min). (**G**) Temperature profiles of protoplast suspensions at concentrations ranging from 10^5^ to 10^9^ CFU/mL under 808 nm NIR irradiation at 1.5 W/cm^2^. (**H**) Temperature profiles of protoplast suspensions (10^8^ CFU/mL) under 808 nm NIR irradiation at power densities ranging from 0.5 to 3.0 W/cm^2^. (**I**) Photothermal cycling temperature profiles of protoplast suspensions under 808 nm NIR irradiation at 1.5 W/cm^2^. (**J**) Photographs and (**K**) quantification of surviving *E. coli* and *S. epidermidis* colonies following treatment with PBS, protoplasts or protoplasts combined with NIR irradiation (P&N). Data are presented as mean ± SD (*n* = 5). *P < 0.05, **P < 0.01, ***P < 0.001, ****P < 0.0001; ns, not significant.

The optimal lysozyme concentration and treatment duration were first determined through systematic screening. Results indicated that 1 mg/mL lysozyme applied for 1 h represented the optimal extraction conditions (Supplementary Figure S1A–D). Under these conditions, the bacterial cell wall was effectively removed, whereas excessively prolonged treatment duration or elevated lysozyme concentration led to protoplast disruption. SEM and TEM characterization revealed that, compared with intact *R. palustris*, protoplasts exhibited a tendency toward spherical morphology, lacked flagella and showed a more uniform distribution of intracellular contents (Figure 1A;  Supplementary Figure S1E). The particle size of protoplasts prepared by this method ranged from 0.7 to 1.2 μm (Figure 1B; Supplementary Figure S1F), which was smaller than that of intact bacteria (2–3 μm). This size reduction is attributed to the loss of cell wall support, which renders protoplasts susceptible to morphological deformation, transitioning from rod-shaped to spherical, thereby resulting in a decreased diameter. Cell wall fluorescence labeling confirmed successful cell wall removal, as evidenced by a significant reduction in fluorescence intensity in protoplasts compared with intact bacteria (Supplementary Figure S1G). The protoplast extraction yield was calculated to be approximately 90.6%.

Endotoxin quantification demonstrated that the endotoxin level of protoplasts was significantly reduced from approximately 400 EU/L to 30 EU/L compared with intact bacteria (Figure 1C). The differences in chemical composition between *R. palustris* and protoplasts were subsequently analyzed. UV-visible absorption spectroscopy revealed that the absorption spectra of protoplasts and *R. palustris* were nearly identical, with characteristic absorption peaks at 375–590 nm, 800–810 nm and 830–890 nm (Figure 1D), corresponding to the absorption bands of carotenoids (375–590 nm) and BChl a (800–810 nm and 830–890 nm) [25], confirming the presence of these photosynthetic pigments in both samples. Furthermore, comparative analysis of photosynthetic pigment contents revealed that BChl a and carotenoid levels were not significantly different in protoplasts compared with intact bacteria (Supplementary Figure S1H and I). Notably, trace amounts of BChl a and carotenoids were detectable in the supernatant of protoplast suspensions, consistent with the absence of the cell wall barrier (Figure 1E). The lack of the cell wall in protoplasts allows BChl a to be more directly exposed to NIR irradiation, thereby enhancing photothermal conversion efficiency.

### Optimization of photothermal conditions and antibacterial performance of protoplasts

Comparison of the photothermal performance between protoplasts and intact bacteria revealed that protoplasts achieved approximately 25.0% greater temperature elevation under identical irradiation conditions. Upon NIR irradiation, protoplast suspensions were heated from room temperature (25°C) to 45°C, achieving a temperature rise of 20°C, whereas intact bacteria only reached 40°C, corresponding to a temperature rise of 15°C, and the PBS group showed no significant temperature change. (Figure 1F; Supplementary Figure S1J). This difference is attributed to the absence of the cell wall in protoplasts, which allows BChl a to be more directly exposed to NIR irradiation, thereby enhancing photothermal conversion.

The photothermal conversion capability of protoplasts was systematically evaluated under varying concentration and NIR light power density conditions. Results demonstrated that both increasing protoplast concentration and NIR light power density contributed to a progressive enhancement of the photothermal effect (Figure 1G and H). At a concentration of 10^8^ CFU/mL and an irradiation power density of 1.5 W/cm^2^, protoplasts achieved temperatures within the effective bactericidal range (45–55°C) [38, 39]. Based on these findings, this concentration and power density were selected for subsequent photothermal treatment experiments.

Photothermal cycling tests demonstrated that protoplasts consistently reached 45°C across three successive irradiation cycles, with no significant changes in photosynthetic pigment content observed before and after irradiation (Figure 1I; Supplementary Figure S1M and N). TEM imaging further revealed that NIR irradiation and prolonged immersion triggered the release of bioactive components from protoplasts (Supplementary Figure S1O). Additionally, TEM observations showed that protoplasts stored in sterile water underwent fragmentation, whereas those maintained in PBS retained structural integrity. However, partial release of intracellular contents was observed in PBS-stored protoplasts, as evidenced by the decreased electron density at the center of protoplasts in TEM images (Supplementary Figure S1O). Collectively, these results demonstrate that protoplasts maintain stable photothermal performance under repeated NIR irradiation, while their bioactive contents are gradually released over time and upon NIR exposure. This dual behavior renders protoplasts particularly suited for wound therapy: during the inflammatory phase, NIR-induced heating enables effective bacterial eradication, whereas during the proliferative phase, the sustained release of bioactive components facilitates wound healing.

The antibacterial efficacy of protoplasts against *E. coli* and *S. epidermidis* was subsequently evaluated. Under NIR irradiation, the P&N group demonstrated significant inhibition of bacterial growth against both strains. Specifically, *E. coli* colony counts decreased from 21.41 × 108 to 5.27 × 10^8^ CFU, and *S. epidermidis* counts decreased from 22.99 × 108 to 4.7 × 10^8^ CFU, corresponding to inhibition rates of 75.4% and 79.4%, respectively (Figure 1J and K).

### Preparation, characterization and stability evaluation of P@G

To prepare P@G, the optimal ionic crosslinking conditions for sodium alginate hydrogel were first established. A calcium chloride concentration of 0.5% (w/v) was identified as the optimal crosslinker concentration. Hydrogels crosslinked at 0.2–0.4% (w/v) exhibited excessive fluidity and failed to maintain gel integrity, whereas at 0.5% (w/v), the hydrogel maintained intact morphology (Supplementary Figure S2A). Rheological analysis confirmed that the blank hydrogel 0.5% (w/v) maintained G′ > G″ across varying frequency (1–100 rad/s), temperature (25–70°C) and strain (0.1–20%) ranges (Supplementary Figure S2C and D), confirming its solid-like viscoelastic behavior and mechanical stability. Therefore, 0.5% (w/v) calcium chloride was selected as the optimal crosslinking concentration for subsequent hydrogel preparation.

**Figure 2:**
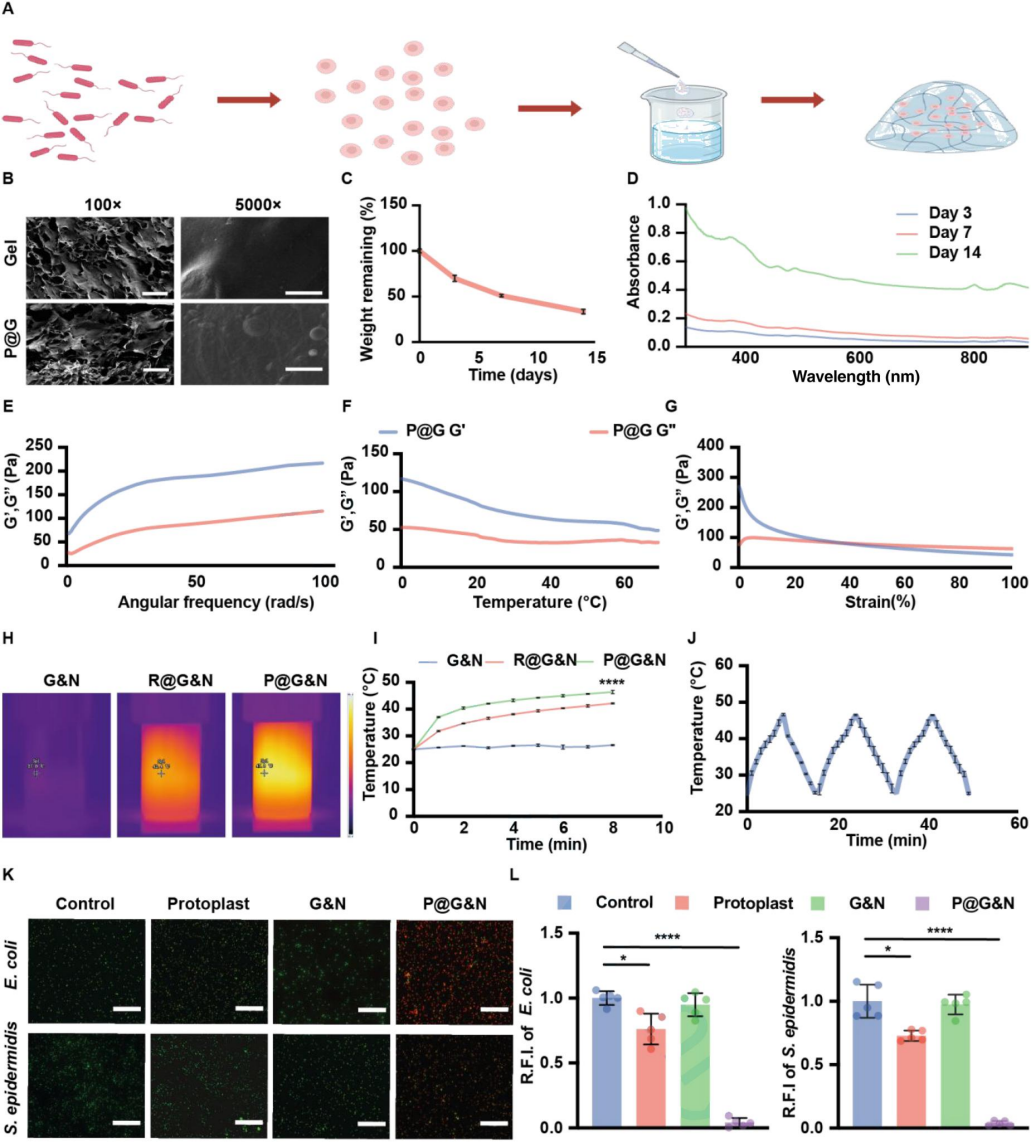
Fabrication, characterization and photothermal antibacterial performance of P@G. (**A**) Schematic illustration of the P@G fabrication process (created with BioRender.com). (**B**) SEM micrographs of gel and P@G at 100× magnification (scale bar: 100 μm) and 5000× magnification (scale bar: 3 μm). (**C**) Weight remaining of P@G during 14-day degradation in PBS under continuous shaking at 100 rpm and 37°C. (**D**) Absorbance values of supernatants collected from P@G on Days 3, 7 and 14 during incubation in PBS under continuous shaking at 100 rpm and 37°C. (**E**) Angular frequency sweep of P@G at 37°C and 1.0% strain. (**F**) Temperature sweep of P@G at an angular frequency of 10 rad/s and 1.0% strain. (**G**) Strain sweep of P@G at 37°C and an angular frequency of 10 rad/s over a strain range of 0.1–100%. (**H**) Infrared thermal images and (**I**) temperature profiles of G&N, R@G&N and P@G&N under 808 nm NIR irradiation at 1.5 W/cm^2^ for 8 min. (**J**) Photothermal cycling temperature profiles of P@G&N under 808 nm NIR irradiation at 1.5 W/cm^2^. (**K**) Live/dead fluorescence staining images and (**L**) R.F.I. of *E. coli* and *S. epidermidis* following treatment with PBS, protoplasts, G&N and P@G&N. Scale bar: 75 μm. Data are presented as mean ± SD (*n* = 5). *P < 0.05, **P < 0.01, ***P < 0.001, ****P < 0.0001; ns, not significant.

Following optimization of crosslinking conditions, P@G was fabricated according to the established procedure (Figure 2A). Upon incorporation of protoplasts, the hydrogel acquired a pale pink color similar to that of *R. palustris* suspensions (Supplementary Figure S2B). At 100× magnification, no significant morphological difference was observed between P@G and blank gel; however, at 5000× magnification, the surface of blank gel appeared smooth, while P@G displayed surface protrusions corresponding to the encapsulated protoplasts (Figure 2B). The EE of protoplasts within P@G was subsequently calculated to be 93.2% based on the established standard curve and corresponding formula (Supplementary Figure S2K).

To evaluate the stability of P@G, the remaining weight of P@G was measured over 14 days in PBS at 37°C. P@G degraded gradually, with approximately 30.0% mass loss by Day 3, 49.0% by Day 7 and 66.6% by Day 14 (Figure 2C). Analysis of the degradation supernatants collected on Days 3, 7 and 14 revealed characteristic absorption peaks at 400–500 nm, 800–810 nm and 830–890 nm, indicating the sustained release of carotenoids and BChl a from P@G over time (Figure 2D). SEM imaging on Days 0, 7 and 14 showed that pore size increased progressively during degradation, while the three-dimensional network structure was maintained (Supplementary Figure S2F). Additionally, SEM images obtained on Days 0, 7 and 14 confirmed that protoplasts remained stably embedded within the hydrogel across all time points, demonstrating their long-term structural stability within the hydrogel matrix (Supplementary Figure S2F).

Swelling analysis revealed that P@G exhibited a swelling ratio of approximately 30.0% (Supplementary Figure S2G), a 3.0% increase compared with the hydrogel alone, indicating that P@G can absorb wound exudate through swelling during the treatment period. Together, these results demonstrate that P@G enables the sustained release of protoplasts and their bioactive components through gradual degradation, while maintaining the capacity to absorb wound exudate via swelling. Rheological analysis confirmed that P@G maintained G′ > G″ across varying frequency (1–100 rad/s), temperature (25–70°C) and strain (0.1–20%) ranges (Figure 2E–G), confirming its mechanical stability.

### Photothermal performance and *in vitro* antibacterial activity of P@G&N

To evaluate the photothermal performance of P@G, samples were irradiated with 808 nm NIR light and temperature changes were recorded using a thermal imaging camera. P@G&N reached 47°C within 8 min of irradiation, with the temperature of P@G&N significantly higher than that of R@G&N and G&N (Figure 2H and I). P@G&N exhibited a temperature approximately 5°C higher than R@G&N, indicating superior photothermal conversion efficiency. In contrast, R@G&N only reached approximately 42°C, falling below the effective bactericidal temperature range of 45–55°C, suggesting its photothermal antibacterial capacity may be limited compared with P@G&N.

Photothermal cycling experiments were subsequently conducted to assess the photothermal stability of P@G. Under multiple rounds of 808 nm NIR irradiation, the temperature elevation amplitude remained stable, consistently reaching 47°C throughout all cycles (Figure 2J). Furthermore, P@G that had undergone 14 days of degradation was subjected to NIR irradiation and still reached temperatures above 45°C within 8 min (Supplementary Figure S2H). This is attributed to the retention of protoplasts within the partially degraded hydrogel matrix (Supplementary Figure S2F), which maintained the photothermal conversion capability of P@G. These results demonstrate that P@G can stably encapsulate protoplasts and retain photothermal performance over time.

The *in vitro* antibacterial activity of P@G&N was further evaluated through bacterial plating assays and live/dead staining. Bacterial plating results showed that the number of *S. epidermidis* and *E. coli* colonies was significantly reduced in the P@G&N treatment group compared with controls (Supplementary Figure S2I and J). Live/dead staining assays further confirmed the antibacterial efficacy of P@G&N, with relative fluorescence intensity (R.F.I.) decreased by 95.9% for *E. coli* and 96.2% for *S. epidermidis* (Figure 2K and L), collectively confirming the potent *in vitro* antibacterial activity of P@G&N.

### *In vitro* biocompatibility of protoplasts and P@G&N

**Figure 3:**
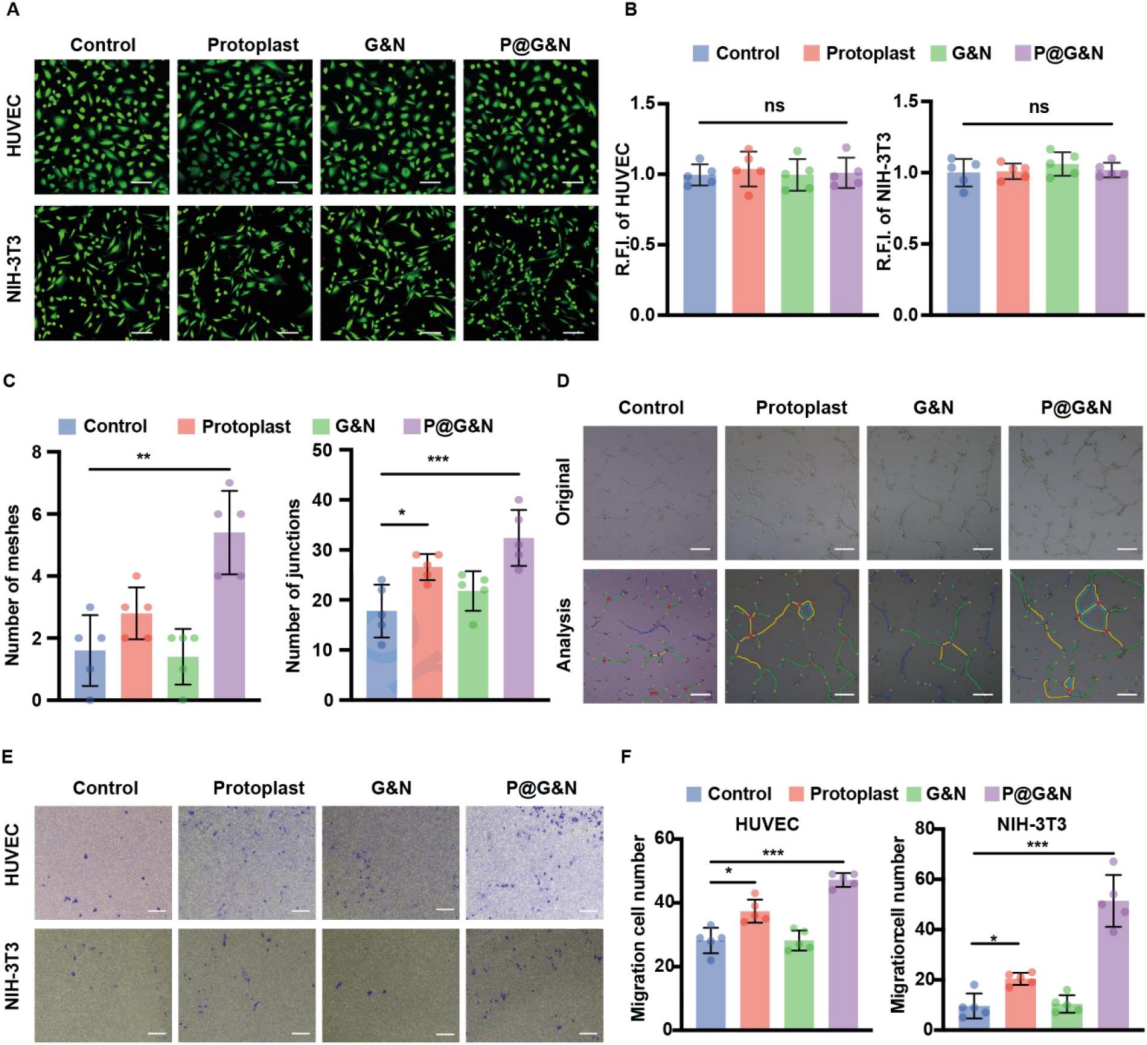
*In vitro* biocompatibility, pro-angiogenic and pro-migratory effects of P@G&N. (**A**) Representative fluorescence images of live/dead staining of HUVECs and NIH-3T3 cells co-cultured with various samples. Scale bar: 200 μm. (**B**) Quantitative R.F.I. from the live/dead cell viability assay. (**C**) Quantification of mesh count and junction count from the *in vitro* tube formation assay. (**D**) Representative images of HUVEC tube formation. Scale bar: 100 μm. (**E**) Optical micrographs and (**F**) quantification of migrated HUVECs and NIH-3T3 cells from the transwell migration assay. Scale bar: 200 μm. Data are presented as mean ± SD (*n* = 5). *P < 0.05, **P < 0.01, ***P < 0.001, ****P < 0.0001; ns, not significant.

A hemolysis assay was performed to evaluate the hemocompatibility of protoplasts at varying concentrations (10^5^–10^9^ CFU/mL). The deionized water group exhibited obvious hemolysis, whereas protoplasts across all tested concentrations showed no significant difference from the saline control, demonstrating excellent hemocompatibility without hemolytic activity (Supplementary Figure S3E). To evaluate the biocompatibility of P@G&N, P@G was first irradiated with 808 nm NIR light and subsequently incorporated into cell culture media, and the growth of NIH-3T3 and HUVEC cells was observed. Live/dead staining assays revealed no significant differences in cell viability between the P@G&N-treated group and the control group, indicating excellent biocompatibility and minimal cytotoxicity (Figure 3A and B).

The effect of P@G&N on cell proliferation was further assessed using a CCK-8 assay. Cell growth was monitored over 48 h, and an additional 24 h assessment was performed at triple the standard dose. No significant differences in cell viability were observed across all groups under either condition (Supplementary Figure S3A–D), indicating that P@G&N had no significant effect on the proliferation of HUVEC and NIH-3T3 cells within 48 h, even at triple the standard dose, confirming the absence of significant cytotoxicity.

Given that wound healing relies on angiogenesis, a tube formation assay was conducted to investigate the pro-angiogenic potential of P@G&N. P@G&N treatment enhanced HUVEC tube formation, as evidenced by increased junctions, meshes and overall angiogenic structures compared with the control group (Figure 3C and D).

Furthermore, a transwell migration assay was performed to evaluate the effect of P@G&N on cell migration. P@G&N significantly promoted the migration of both HUVEC and NIH-3T3 cells, with a markedly higher number of migrated cells observed in the P@G&N group compared with controls (Figure 3E and F).

These results collectively demonstrate that P@G&N exhibits excellent biocompatibility while actively promoting angiogenesis and cell migration, supporting its potential for wound healing applications.

### *In vivo* antibacterial and wound healing-promoting performance of P@G&N

**Figure 4:**
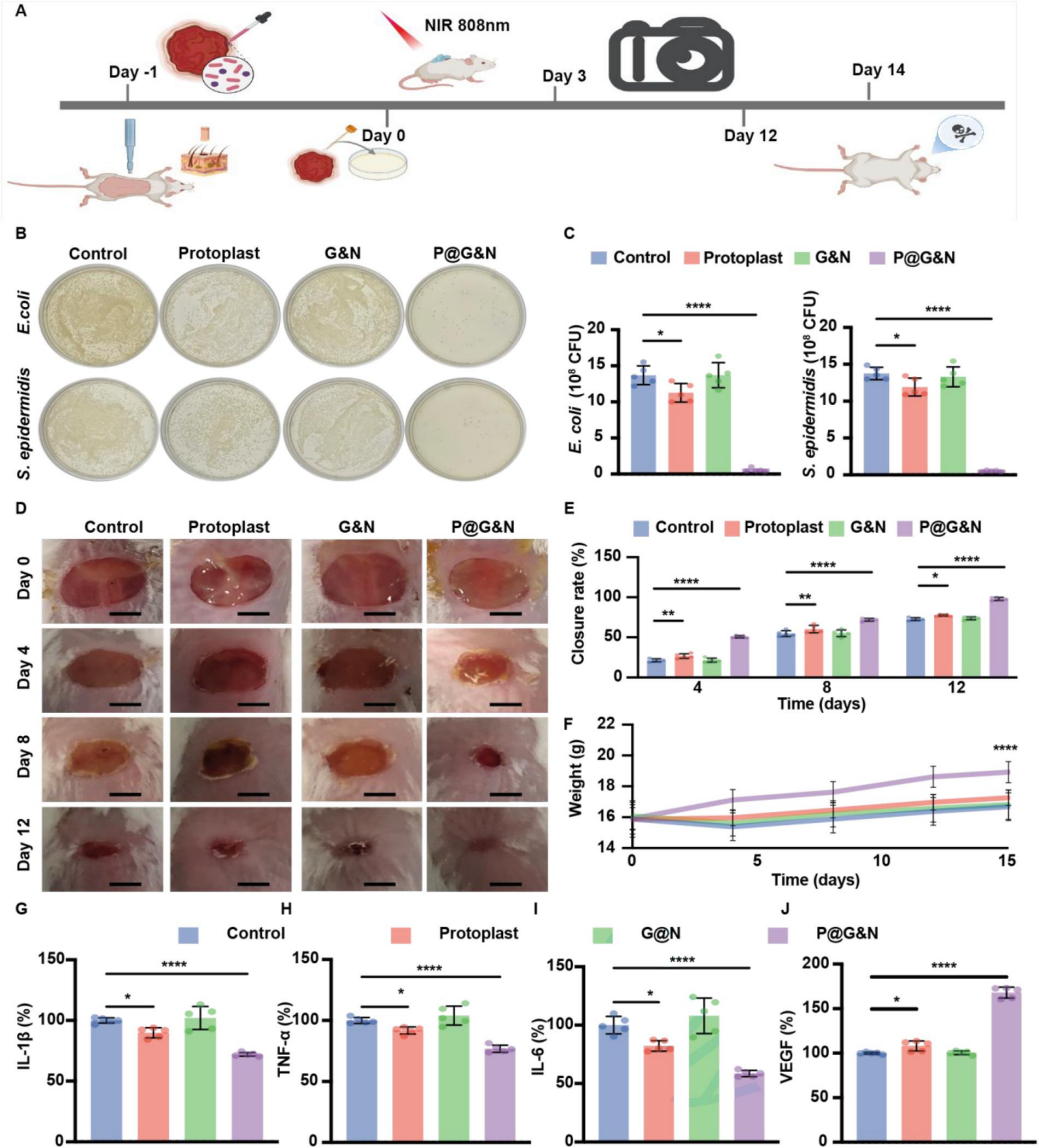
*In vivo* antibacterial and wound healing efficacy of P@G&N. (**A**) Schematic illustration of the animal experimental protocol (created with BioRender.com). (**B**) Photographs of agar plates and (**C**) quantification of surviving *E. coli* and *S. epidermidis* colonies isolated from wound tissue homogenates following treatment with PBS, protoplasts, G&N and P@G&N. (**D**) Representative wound photographs of mice from each group over a 12-day healing period. Scale bar: 5 mm. (**E**) Dynamic changes in wound closure rates across treatment groups over a 12-day period. (**F**) Body weight changes in mice following different treatments. (**G**) Serum concentrations of IL-1β, (**H**) TNF-α, (**I**) IL-6 and (**J**) VEGF in mice from the PBS, protoplast, G&N and P@G&N groups on Day 3. Data are presented as mean ± SD (*n* = 5). *P < 0.05, **P < 0.01, ***P < 0.001, ****P < 0.0001; ns, not significant.

The workflow of the animal experiment is illustrated in Figure 4A. To establish an infected wound model, a 0.8 cm wound defect was created on each mouse, followed by inoculation with *S. epidermidis* and *E. coli*. On Day 0, purulent exudation was observed at the wound site, and bacterial culture results confirmed the successful establishment of the infection model (Supplementary Figure S3F and G).

Mice were subsequently subjected to treatment. Infrared thermal imaging of wound sites revealed that, under 808 nm NIR irradiation, different treatment groups exhibited varying degrees of photothermal response. The P@G&N group showed the most pronounced temperature elevation, with wound surface temperature rapidly rising from baseline body temperature (approximately 36°C) to 53.5°C, significantly higher than that of all other groups. Notably, although the *in vivo* baseline wound temperature (approximately 36°C) was higher than the ambient temperature used *in vitro* (25°C), P@G&N still demonstrated effective photothermal heating capacity. Following each treatment, wound sites were covered with a transparent medical dressing film to ensure retention of the applied materials on the wound surface (Supplementary Figure S3J).

Subsequent therapeutic intervention results demonstrated that the P@G&N group achieved significantly superior *in vivo* bactericidal efficacy compared with all other groups (Figure 4B and C). Under 808 nm NIR irradiation, the *E. coli* colony count at the wound site decreased from 1.36 × 10^9^ CFU to 3.88 × 10^7^ CFU, and the *S. epidermidis* count decreased from 1.37 × 10^9^ CFU to 4.60 × 10^7^ CFU, yielding bactericidal rates of 97.2% and 96.7% against *E. coli* and *S. epidermidis*, respectively, demonstrating that P@G&N achieves highly efficient *in vivo* photothermal bactericidal activity.

To comprehensively assess the wound healing efficacy of P@G&N, wound photographs were captured and body weight was recorded at predetermined time intervals. Wound photographs taken on Days 0, 4, 8 and 12 demonstrated that the P@G&N group exhibited significantly superior wound healing compared with the control group (Figure 4D). Specifically, the P@G&N group achieved a wound closure rate of approximately 50.8% on Day 4, which further increased to 97.9% by Day 12, surpassing the control group by 25.2% by Day 12 (Figure 4E). No body weight loss was recorded in the P@G&N group throughout the experimental period (Figure 4F), indicating the absence of systemic toxicity. Notably, the protoplast group also demonstrated a certain degree of enhanced wound healing compared with the control group, which may be attributed to the release of bioactive components such as carotenoids from protoplasts into the wound site, thereby reducing local free radical levels and facilitating wound recovery [40, 41].

To evaluate the effects of different treatments on the inflammatory response during the early phase of wound infection, serum concentrations of pro-inflammatory cytokines IL-1β, TNF-α and IL-6 were quantified in mice from each group on Day 3 post-treatment. Compared with the control group, the P@G&N group showed the most significant reductions in IL-1β, TNF-α and IL-6 levels, indicating that P@G&N effectively suppresses the systemic inflammatory response during the early phase of infection (Figure 4G–I). Serum VEGF concentrations were further quantified on Day 3 post-treatment to characterize the angiogenic regulatory effects of P@G&N. The P@G&N group exhibited significantly higher serum VEGF levels than all other groups, demonstrating that P@G&N can upregulate VEGF secretion and promote neovascularization (Figure 4J).

### *In vivo* wound healing and immunomodulatory mechanisms of P@G&N

**Figure 5:**
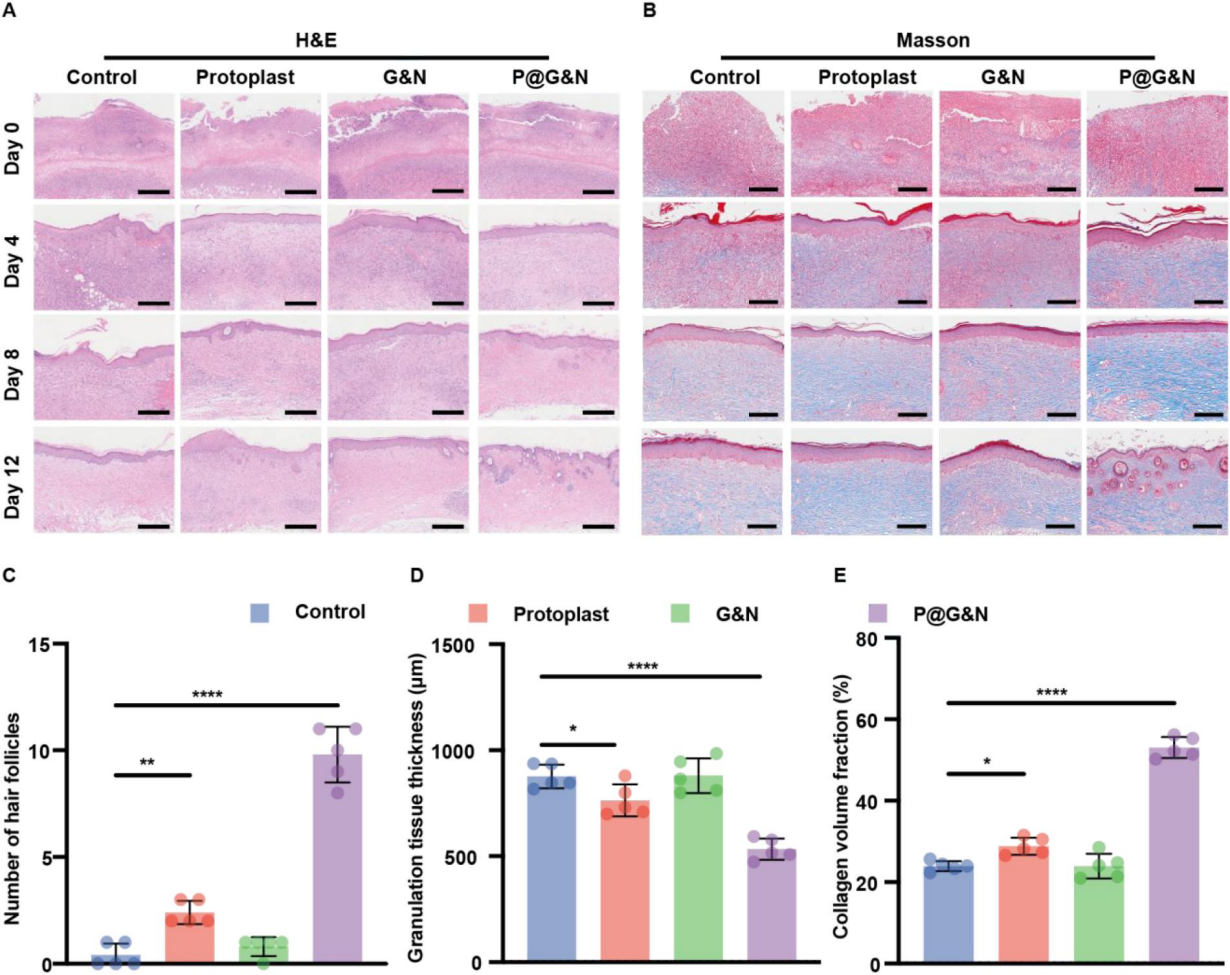
Histological analysis of wound tissue repair. (**A**) H&E staining of wound tissue sections harvested on Days 0, 4, 8 and 12. Scale bar: 300 μm. (**B**) Masson’s trichrome staining of wound tissue sections harvested on Days 0, 4, 8 and 12. Scale bar: 100 μm. (**C**) Quantification of hair follicle count in H&E-stained wound sections on Day 12 across treatment groups. (**D**) Quantification of granulation tissue thickness in H&E-stained wound sections on Day 12 across treatment groups. (**E**) Quantification of collagen area fraction in Masson’s trichrome-stained wound sections on Day 12 across treatment groups. Data are presented as mean ± SD (*n* = 5). *P < 0.05, **P < 0.01, ***P < 0.001, ****P < 0.0001; ns, not significant.

To elucidate the histological mechanisms underlying the differential wound healing efficiency across treatment groups, wound tissue sections collected at multiple time points were subjected to pathological analysis. H&E staining (Figure 5A) revealed that, compared with the control group, the P@G&N group exhibited markedly reduced inflammatory cell infiltration and early granulation tissue formation as early as Day 4. By Day 8, prominent cell migration and tissue proliferation were observed. By Day 12, complete re-epithelialization had been achieved, with hair follicle reconstruction and near-complete dermal tissue regeneration.

Quantitative analysis demonstrated that the P@G&N group had approximately 10 hair follicles on Day 12, significantly more than all other groups, and a granulation tissue thickness of approximately 500 μm, significantly less than that of the control group (approximately 800 μm), indicating that this treatment effectively promotes tissue structural remodeling at the wound site (Figure 5C and D).

Masson’s trichrome staining (Figure 5B) revealed that collagen deposition in the P@G&N group was higher than that of the other three groups at all time points. Quantitative analysis further confirmed that the collagen area fraction in the P@G&N group on Day 12 was approximately three times that of the control group and significantly superior to all other treatment groups (Figure 5E), demonstrating that P@G&N significantly promotes collagen synthesis and orderly deposition. Furthermore, H&E staining of wound tissues at 0 and 24 h post-treatment revealed no detectable thermal damage in the P@G&N group, confirming the safety of P@G&N treatment (Supplementary Figure S3L).

**Figure 6:**
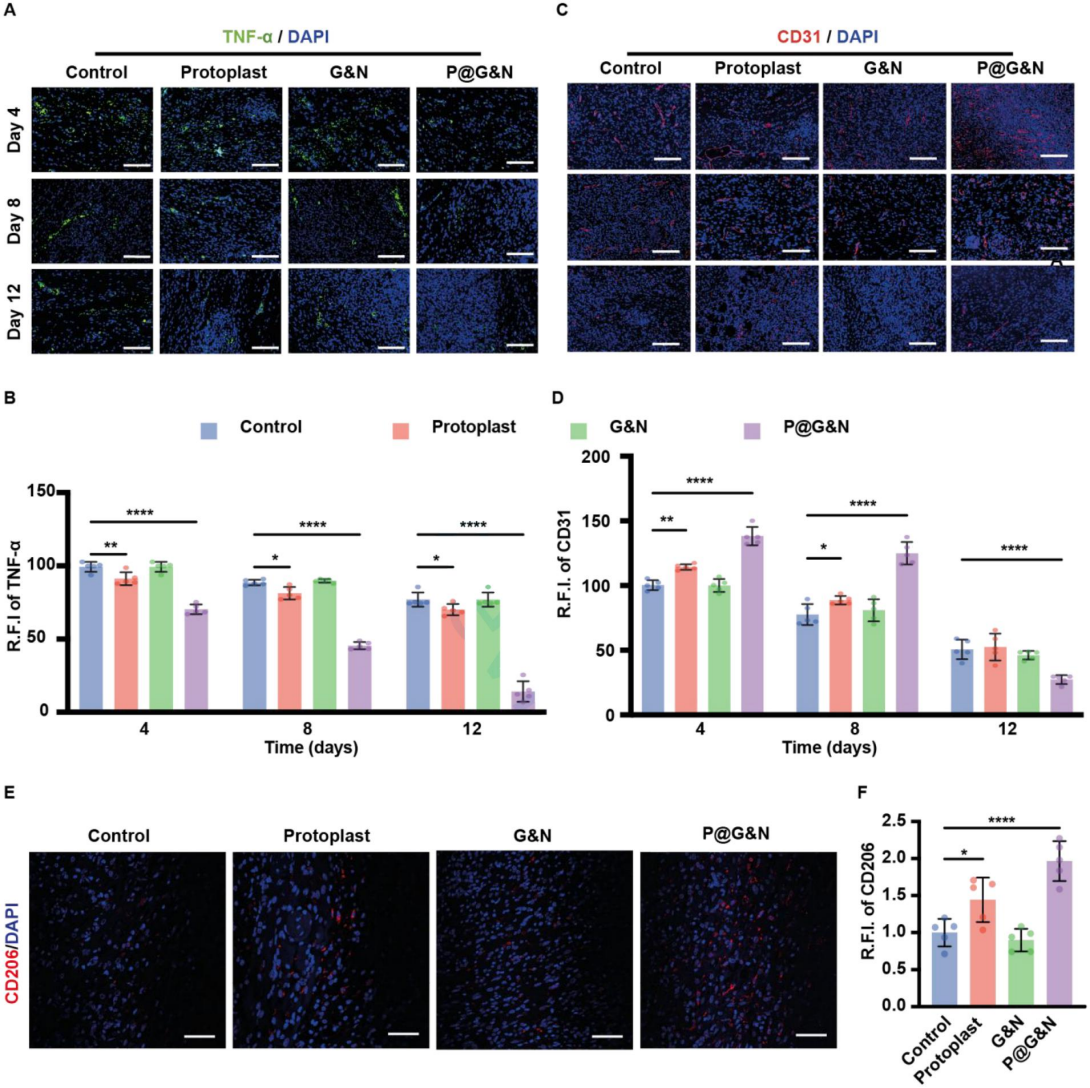
Immunofluorescence analysis of TNF-α, CD31 and CD206 expression in wound tissues. (**A**) Immunofluorescence staining of TNF-α in wound tissue sections on Days 4, 8 and 12. Scale bar: 100 μm. (**B**) Quantitative analysis of relative TNF-α fluorescence intensity. (**C**) Immunofluorescence staining of CD31 in wound tissue sections on Days 4, 8 and 12. Scale bar: 100 μm. (**D**) Quantitative analysis of relative CD31 fluorescence intensity. (**E**) Immunofluorescence staining of CD206 in wound tissue sections on Day 4. Scale bar: 50 μm. (**F**) Quantitative analysis of relative CD206 fluorescence intensity across treatment groups on Day 4. Data are presented as mean ± SD (*n* = 5). *P < 0.05, **P < 0.01, ***P < 0.001, ****P < 0.0001; ns, not significant.

To further investigate the underlying wound healing mechanisms, immunofluorescence staining of TNF-α and CD31 was performed. TNF-α staining revealed that the P@G&N group maintained significantly lower inflammatory cytokine levels throughout the treatment period compared with the control group (Figure 6A and B), suggesting that P@G&N treatment attenuates local inflammatory responses by eradicating bacteria at the wound site.

Quantitative immunofluorescence staining of CD31 revealed that from Day 4 to Day 8, CD31 expression in the wound tissue of the P@G&N group was persistently and significantly higher than that of all other groups (Figure 6C and D), indicating that P@G&N effectively promotes neovascularization and accelerates the vascularization process at the wound site. This finding is corroborated by the significantly elevated serum VEGF levels observed in the P@G&N group on Day 3, suggesting that P@G&N upregulates VEGF secretion to drive new vessel formation, thereby resulting in higher CD31 fluorescence intensity compared with other groups.

To further investigate the effects of P@G&N on the immune microenvironment at the wound site, CD206 immunofluorescence staining was performed on tissue sections collected on day 4. CD206 is a well-established marker of M2 macrophages [42]. Compared with the control group, CD206 expression was significantly increased in the P@G&N-treated group (Figure 6E and F), suggesting that P@G&N promotes M2 macrophage polarization at the wound site. M2 macrophages are known to downregulate pro-inflammatory cytokines, secrete VEGF to promote angiogenesis and facilitate endothelial cell proliferation, fibroblast activation and collagen deposition [43, 44]. These findings are consistent with the observed reductions in serum pro-inflammatory cytokine levels and elevated VEGF concentrations on Day 3, as well as the decreased inflammatory levels, increased collagen deposition and enhanced angiogenesis observed from Day 4 onwards in the treatment group.

Based on these findings, we propose that P@G&N promotes infected wound healing through three synergistic mechanisms. First, NIR irradiation activates BChl a in protoplasts, generating localized hyperthermia that rapidly eliminates bacteria and resolves persistent pro-inflammatory stimulation [15, 45]. Second, NIR irradiation triggers the release of carotenoids from protoplasts within P@G, which scavenge ABTS and DPPH free radicals (Supplementary Figure S1K and L), thereby reducing Reactive oxygen species (ROS) levels at the wound site and preventing infection-driven chronic inflammation [40, 46, 47]. This anti-inflammatory effect promotes macrophage polarization toward the M2 phenotype, which downregulates pro-inflammatory cytokines, secretes VEGF to stimulate angiogenesis and supports endothelial cell proliferation, fibroblast activation and collagen deposition [44]. Consistently, the P@G&N-treated group exhibited elevated serum VEGF levels, reduced inflammatory cytokines, enhanced angiogenesis and increased collagen deposition, collectively contributing to accelerated wound healing.

### *In vivo* biosafety evaluation of P@G&N

To evaluate the *in vivo* safety of P@G&N treatment, serum biochemical analysis and H&E staining of major organs were performed on mice following three rounds of treatment. Serum biochemical analysis revealed that ALT, AST, BUN and CREA concentrations in all treatment groups on Day 14 were within normal reference ranges and showed no statistically significant differences compared with the control group (Supplementary Figure S4A–D). These indices reflect hepatic function (ALT, AST) and renal filtration function (BUN and CREA), respectively, indicating that P@G&N treatment does not adversely affect hepatic or renal function. Furthermore, serum inflammatory cytokine analysis demonstrated that IL-1β, TNF-α and IL-6 levels in the P@G&N group on Day 14 were comparable to those of healthy control mice, with no statistically significant differences observed (Supplementary Figure S4E–G), confirming that P@G&N treatment does not cause long-term perturbation of immune homeostasis. H&E staining of major organs (heart, liver, spleen, lung and kidney) revealed no observable histological abnormalities across all treatment groups (Supplementary Figure S4H), demonstrating that P@G&N does not induce tissue damage to major organs and exhibits excellent systemic biocompatibility.

To investigate the metabolic fate of P@G&N, protoplasts were labeled with DSPE-PEG-Rhodamine and loaded into the hydrogel for fluorescence tracking. The hydrogel degraded completely within 7 days at the wound site (Supplementary Figure S4I), and the protoplasts were gradually cleared through the heart, lungs, liver and kidneys within 7 days (Supplementary Figure S4J), confirming systemic metabolic clearance without organ accumulation.

In conclusion, P@G&N demonstrates excellent *in vivo* biocompatibility and systemic clearance, with no hepatic, renal or immunological toxicity observed, and with complete metabolic clearance within 7 days.

## Discussion

The construction of the P@G&N system is guided by three core design principles: the multifunctional properties of protoplasts, the carrier advantages of the sodium alginate hydrogel and the synergistic therapeutic effects of photothermal therapy in infected wounds. As the central bioactive component, protoplasts provide several key advantages. First, they exhibit enhanced photothermal conversion efficiency. Removal of the cell wall eliminates a barrier to light absorption, enabling direct exposure of photosynthetic pigments to the near-infrared (NIR) light field and resulting in 25.0% higher photothermal conversion efficiency compared with intact bacteria. Second, protoplasts present a markedly reduced endotoxin burden. LPS, a major component of the bacterial outer membrane, is the primary source of endotoxins; its removal reduces endotoxin levels from ∼400 EU/L to ∼30 EU/L, significantly improving biosafety. Third, protoplasts demonstrate enhanced antioxidant and anti-inflammatory potential. They retain the full spectrum of bioactive components from photosynthetic bacteria, including carotenoids, while the absence of the cell wall facilitates their release into the extracellular environment, thereby increasing bioavailability. In addition, systematic optimization identified the optimal lysozyme concentration for protoplast preparation, and their temporal, photothermal and osmotic stability were thoroughly validated. These findings provide a solid foundation for scalable production and future clinical translation.

Sodium alginate, a natural polysaccharide-derived biomaterial, exhibits excellent biocompatibility and wound moisturizing capacity, making it a suitable carrier for wound therapy [48]. Incorporating protoplasts into the sodium alginate hydrogel not only enhances protoplast stability but also facilitates the enrichment and sustained release of bioactive components at the wound site, thereby improving overall therapeutic efficacy.

Mechanistically, P@G&N promotes infected wound healing through a coordinated, multi-modal process. NIR-induced hyperthermia enables rapid bacterial eradication, while the release of carotenoids reduces free radical levels and alleviates oxidative stress. Concurrently, sustained protoplast activity modulates the wound immune microenvironment by promoting M2 macrophage polarization, leading to the downregulation of pro-inflammatory cytokines, enhanced angiogenesis and improved collagen deposition. Together, these effects synergistically accelerate tissue regeneration and wound repair.

Nevertheless, several limitations of this study warrant acknowledgment and should be addressed in future work. First, the infection model involved only two common cutaneous pathogens, *E. coli* and *S. epidermidis*. The therapeutic efficacy of P@G&N has not yet been validated in diabetic wound models or in infection models involving clinically prevalent drug-resistant pathogens such as methicillin-resistant *Staphylococcus aureus* (MRSA), which to some extent limits the generalizability of the present findings and their clinical translation potential. Second, the specific molecular pathways underlying P@G&N-mediated wound healing promotion remain incompletely elucidated. Future studies will employ RNA sequencing or protein arrays to further dissect the mechanistic basis of its pro-healing effects. Third, although this study identified core compositional differences between protoplasts and intact photosynthetic bacteria, a systematic comparison of other bioactive components has not been conducted. Protoplasts and intact photosynthetic bacteria may differ in the content and bioavailability of functional small molecules, but this remains unclear. The potential contributions of these components to wound healing warrant further investigation. Future studies will aim to address the aforementioned limitations to further advance the clinical translation of P@G&N.

## Conclusion

In summary, this study successfully fabricated a hydrogel dressing with potent antibacterial properties and significant wound healing efficacy. Upon NIR irradiation, P@G&N converts light energy into thermal energy, exhibiting broad-spectrum bactericidal activity against both gram-positive and gram-negative bacteria. Following bacterial eradication, P@G&N significantly downregulates pro-inflammatory cytokines, promotes angiogenesis, facilitates tissue regeneration and collagen deposition, thereby supporting its application in the treatment of infected wounds.

## Supplementary Material

rbag138_Supplementary_Data
