# Supplementary material for: Engineering a cell wall-deficient protoplast-incorporated hydrogel for infected wound healing
Source: Regen Biomater. 2026 Jun 18;13:rbag138. doi: 10.1093/rb/rbag138 (PMC13390284; doi:10.1093/rb/rbag138)
Supplement: rbag138_Supplementary_Data [file rbag138_supplementary_data.docx]

**Engineering a Cell Wall–Deficient Protoplast-Incorporated Hydrogel for Infected Wound Healing**

Yan Zeng^1,2^, Weichao Ding^1,2^, Xiaohan Zhou^2^, Qishan Li^2,3^, Junshu Guo^2^, Yingxian Xiao^2,3^, Rui Zhang^2,3^, Jiacong Ai^2,3^, Junyao Deng^2,3^, Guanmou Li^2^*, Xiaolin Cui^4,5^* and Zhenhua Li^1,2^*

^1^School of Biomedical Engineering, Southern Medical University, Guangzhou, Guangdong 510515, China

^2^The Tenth Affiliated Hospital, Southern Medical University (Dongguan People's Hospital), Dongguan, Guangdong 523059, China

^3^Shenzhen Clinical Medical School, Southern Medical University, Shenzhen, Guangdong 518000, China

^4^School of Medicine, The Chinese University of Hong Kong Shenzhen, Guangdong 518172, China

^5^Joint Laboratory of CUHKSZ-Dalian Practical Biotechnology, The Chinese University of Hong Kong, Shenzhen, Guangdong, 518172, China

*Corresponding authors Email: lanlw@gdyzy.edu.cn (G. Li), stevencui@cuhk.edu.cn (X. Cui), or zhenhuali@hbu.edu.cn (Z. Li)


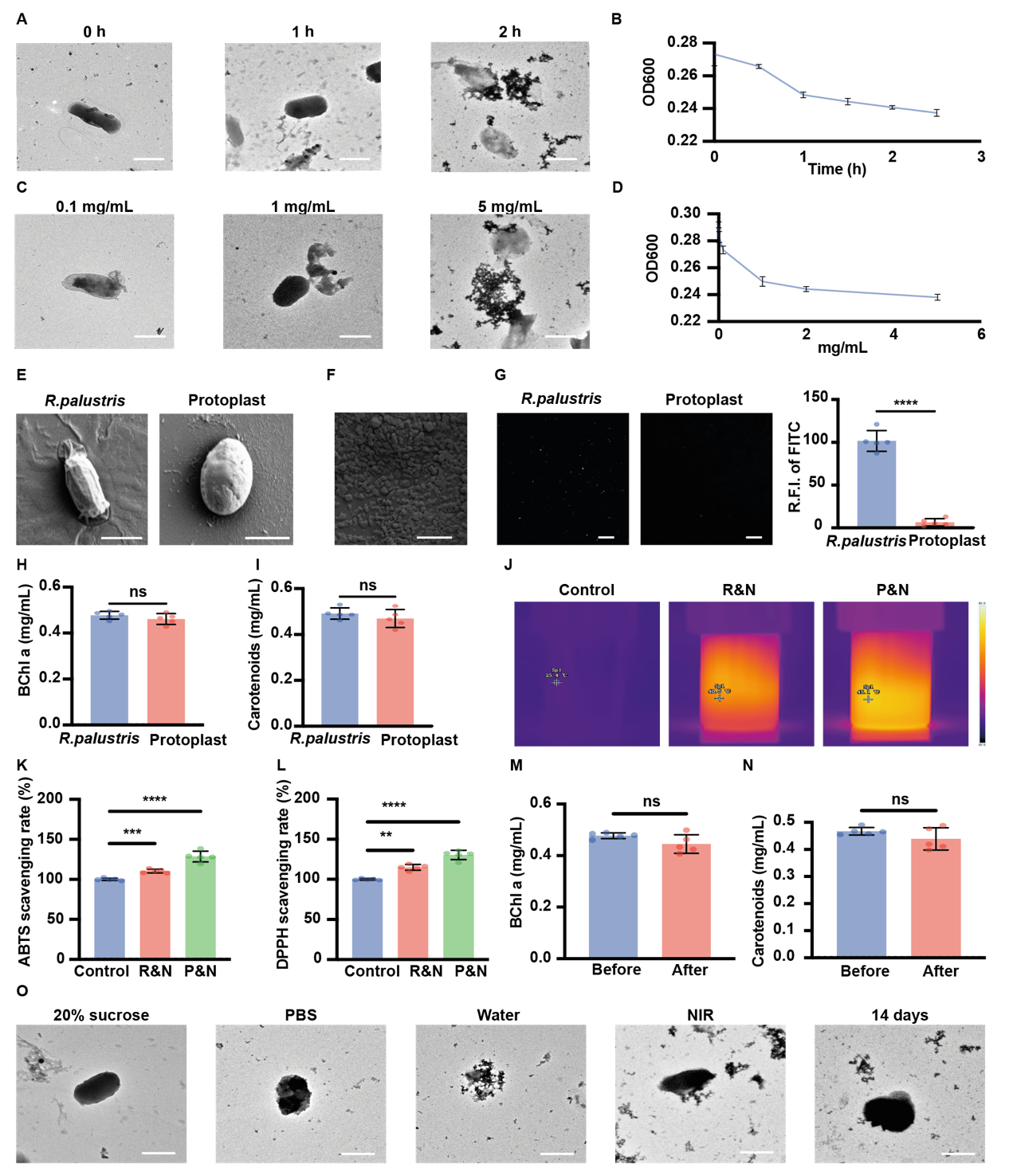


**Figure S1. Preparation and characterization of *R. palustris* protoplasts.** (A) OD₆₀₀ values of *R. palustris* suspensions in sterile water following treatment with 2 mg/mL lysozyme at 0, 0.5, 1, 1.5, 2, and 2.5 h, and (B) corresponding TEM micrographs at representative time points (0, 1, and 2 h). Scale bars: 1 μm. (C) OD₆₀₀ values of *R. palustris* suspensions in sterile water following 1 h treatment with lysozyme at concentrations ranging from 0.01 to 5 mg/mL, and (D) corresponding TEM micrographs of representative groups (0.1, 1, and 5 mg/mL). Scale bars: 1 μm. (E) SEM micrographs of *R. palustris* and its derived protoplasts. Scale bar: 1 μm. (F) SEM micrographs of protoplast particles. Scale bar: 5 μm. (G) Fluorescence microscopy images and relative fluorescence intensity of *R. palustris* and its derived protoplasts. Scale bars: 20 μm. (H) BChl *a* and (I) carotenoid contents in *R. palustris* and its derived protoplasts. (J) Infrared thermal images of *R. palustris* and protoplasts under 808 nm NIR irradiation at 1.5 W/cm² for 8 min. (K) ABTS and (L) DPPH radical scavenging activity of PBS, P&N, and R&N groups. (M) BChl *a* and (N) carotenoid contents of protoplasts before and after NIR irradiation. (O) TEM micrographs of protoplasts in 20% sucrose solution, in PBS, in sterile distilled water, after NIR irradiation, and after storage in PBS for 14 days. Scale bars: 1 μm. Data are presented as mean ± SD (n = 5). *P < 0.05, **P < 0.01, ***P < 0.001, ****P < 0.0001; ns, not significant.


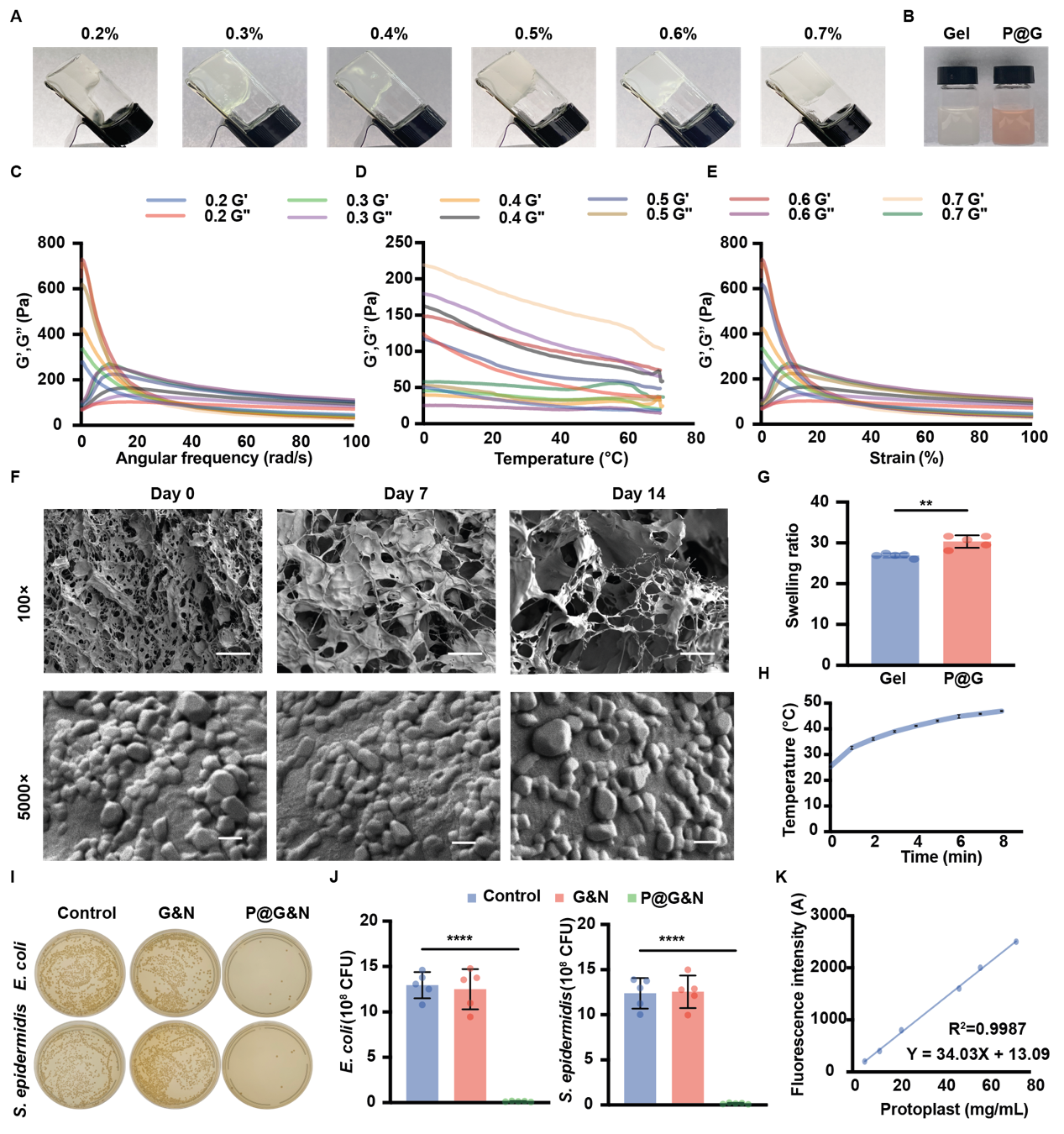


**Figure S2. Optimization and characterization of P@G.** (A) Photographs of sodium alginate hydrogels crosslinked with calcium chloride at varying concentrations (0.2%–0.7%, w/v). (B) Photographs of blank Gel and P@G, showing the pale pink coloration of P@G following protoplast incorporation. (C) Angular frequency sweep of sodium alginate hydrogels crosslinked with calcium chloride at concentrations ranging from 0.2% to 0.7% (w/v) at 37 °C and 1% strain. (D) Temperature sweep of sodium alginate hydrogels crosslinked with calcium chloride at concentrations ranging from 0.2% to 0.7% (w/v) at an angular frequency of 10 rad/s and 1% strain. (E) Strain sweep of sodium alginate hydrogels crosslinked with calcium chloride at concentrations ranging from 0.2% to 0.7% (w/v) at 37 °C and an angular frequency of 10 rad/s over a strain range of 0.1%–100%. Data represent the mean of three independent measurements. (F) SEM micrographs of P@G following immersion in PBS at 37 °C on day 0, day 7, and day 14 at 100× magnification (scale bar: 100 μm) and 5000× magnification (scale bar: 1 μm). (G) Swelling ratios of Gel and P@G at swelling equilibrium in PBS at 37 °C. (H) Photothermal heating curves of P@G following 14-day immersion in PBS at 37 °C under 808 nm NIR irradiation at 1.5 W/cm² for 8 min. (I) Photographs of agar plates and (J) quantification of surviving *E. coli* and *S. epidermidis* colonies following treatment with PBS, G&N, and P@G&N. (K) Standard curve of fluorescence intensity for calculation of protoplast encapsulation efficiency. Data are presented as mean ± SD (n = 5). *P < 0.05, **P < 0.01, ***P < 0.001, ****P < 0.0001; ns, not significant.

**
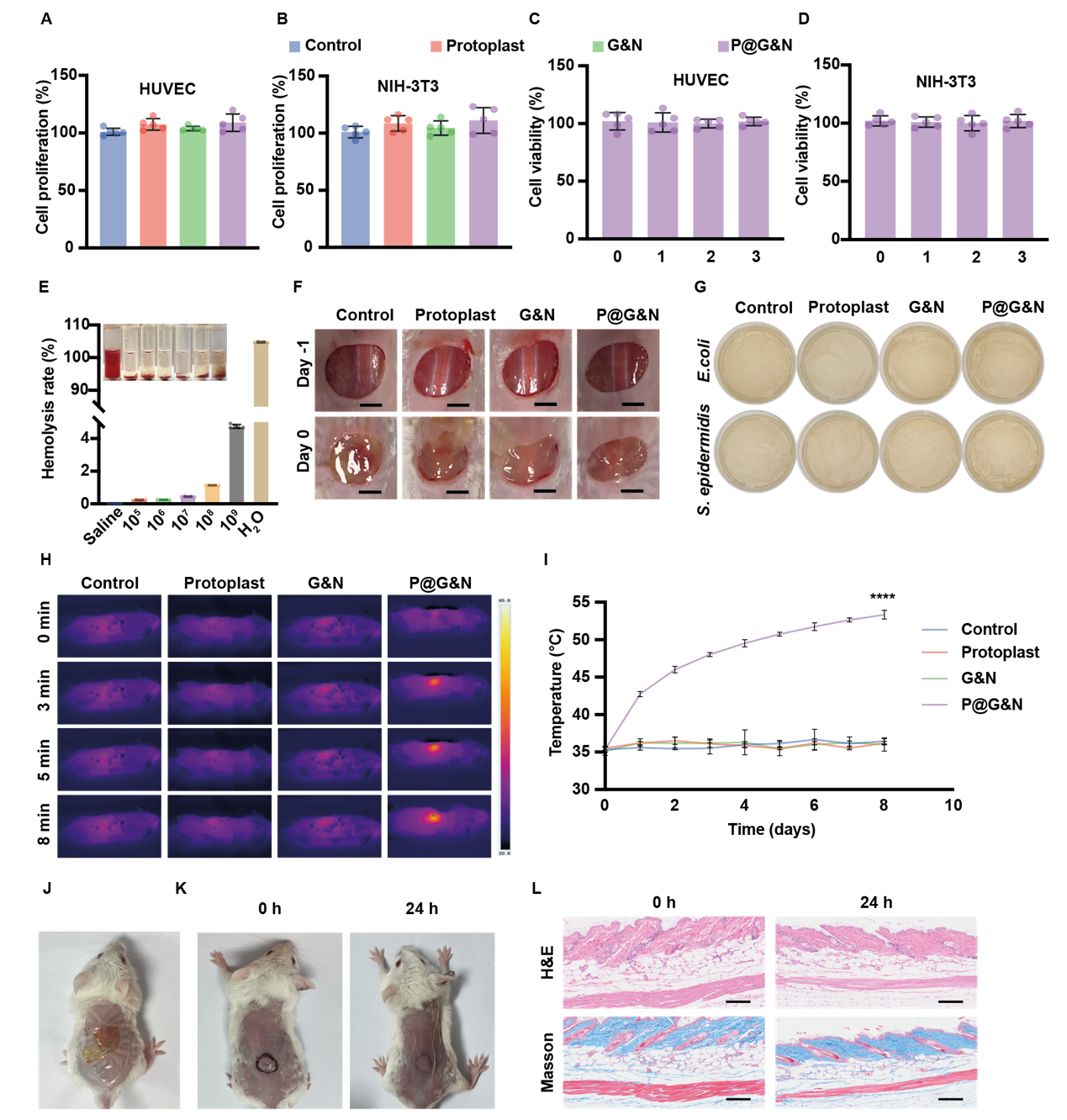
**

**Figure S3. Biocompatibility evaluation and in vivo infection model characterization.** (A) Cell viability of HUVECs and (B) NIH-3T3 cells cultured with P@G&N for 48 h, as assessed by CCK-8 assay. (C) Cell viability of HUVECs and (D) NIH-3T3 cells cultured with P@G&N at 0×, 1×, 2×, and 3× the standard dose for 24 h, as assessed by CCK-8 assay. (E) Quantitative hemolysis rates of protoplasts at varying concentrations. (F) Photographs of infected wound areas in mice following treatment with PBS, protoplasts, G&N, and P@G&N on day 0 prior to treatment initiation. Scale bar: 5 mm. (G) Photographs of agar plates showing bacterial colonies cultured from wound swabs on day 0 prior to treatment initiation, confirming successful establishment of the infection model. (H) Infrared thermal images and (I) temperature profiles of wound sites in mice from the PBS, protoplast, G&N, and P@G&N groups under 808 nm NIR irradiation at 1.5 W/cm². (J) Photographs of wound sites covered with transparent medical dressing film following treatment. (K) Representative photographs of uninfected mice at 0 h and 24 h following P@G&N treatment, and (L) corresponding H&E and Masson's trichrome staining of skin tissue sections at 0 h and 24 h post-irradiation. Scale bar: 100 μm. Data are presented as mean ± SD (n = 5). *P < 0.05, **P < 0.01, ***P < 0.001, ****P < 0.0001; ns, not significant.

**
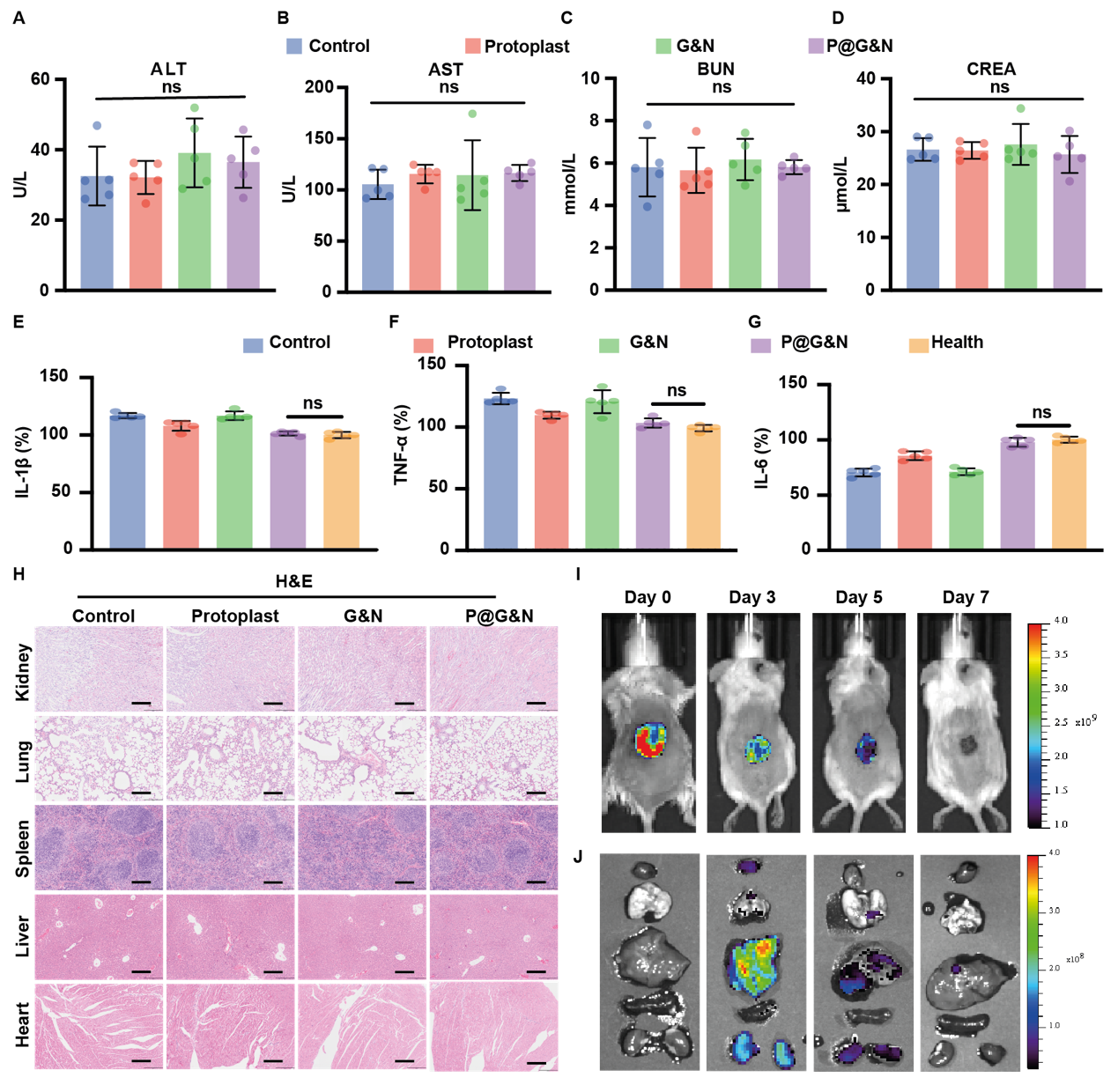
**

**Figure S4. In vivo biosafety assessment and biodistribution of P@G.** (A) Serum concentrations of ALT, (B) AST, (C) BUN, and (D) CREA in mice from each treatment group on day 14 following three rounds of treatment. (E) Relative serum concentrations of IL-1β, (F) TNF-α, and (G) IL-6 in mice from the PBS, protoplast, G&N, and P@G&N groups and healthy mice on day 14 following three rounds of treatment. (H) H&E staining of major organs harvested from each treatment group on day 14 following three rounds of treatment. Scale bar: 100 μm. (I) In vivo fluorescence imaging of wound sites on days 0, 3, 5, and 7 following P@G&N treatment. (J) Ex vivo fluorescence imaging of major organs on days 0, 3, 5, and 7 following P@G&N treatment. Data are presented as mean ± SD (n = 5). *P < 0.05, **P < 0.01, ***P < 0.001, ****P < 0.0001; ns, not significant.
